# Tuning IgE: IgE-Associating Molecules and Their Effects on IgE-Dependent Mast Cell Reactions

**DOI:** 10.3390/cells10071697

**Published:** 2021-07-05

**Authors:** Tomoaki Ando, Jiro Kitaura

**Affiliations:** 1 Atopy (Allergy) Research Center, Juntendo University Graduate School of Medicine, Tokyo 113-8421, Japan; 2 Department of Science of Allergy and Inflammation, Juntendo University Graduate School of Medicine, Tokyo 113-8421, Japan

**Keywords:** IgE, mast cells, basophils, FcεRI, CD23, histamine-releasing factor (HRF), glycosylation, structure, omalizumab

## Abstract

The recent emergence of anti-immunoglobulin E (IgE) drugs and their candidates for humans has endorsed the significance of IgE-dependent pathways in allergic disorders. IgE is distributed locally in the tissues or systemically to confer a sensory mechanism in a domain of adaptive immunity to the otherwise innate type of effector cells, namely, mast cells and basophils. Bound on the high-affinity IgE receptor FcεRI, IgE enables fast memory responses against revisiting threats of venoms, parasites, and bacteria. However, the dysregulation of IgE-dependent reactions leads to potentially life-threatening allergic diseases, such as asthma and anaphylaxis. Therefore, reactivity of the IgE sensor is fine-tuned by various IgE-associating molecules. In this review, we discuss the mechanistic basis for how IgE-dependent mast cell activation is regulated by the IgE-associating molecules, including the newly developed therapeutic candidates.

## 1. Introduction

Immunoglobulin E (IgE) is a unique Ig isotype with the lowest serum concentration, which confers a sensory mechanism of adaptive immunity to the otherwise innate type of effector cells, including mast cells and basophils. Its relatively long half-life (~3 weeks) on the high-affinity IgE receptor FcεRI, together with the minute-order response time, enables protection from the invasive venoms [1] and fast-moving parasites [2] using the ambush strategy. In addition, bacterial invasion through acute injury is reported to be prevented by IgE and mast cells [3]. To fight against these threats, mast cells and basophils degranulate upon recognition of multivalent antigens by IgEs to rapidly release preformed substances, including histamine and proteases, and initiate the de novo production of lipid mediators, cytokines, and chemokines [4,5,6]. However, dysregulation of this potentially harmful reaction leads to allergic diseases, such as anaphylaxis. In addition to the well-established knowledge obtained from animal models, the central role of IgE in allergic diseases in humans has been endorsed by the emergence of anti-IgE therapy by omalizumab, effective against various allergic diseases, including asthma, chronic spontaneous urticaria, nasal polyps, and allergic rhinitis. To overcome allergic diseases, an understanding of the regulation of IgE functions is essential. In this review, we discuss the fine-tuning of IgE functions by various IgE-binding factors, including the emerging drug candidates. The readers interested in the biological or medical significance of IgE-dependent reactions are referred to other excellent reviews [7,8,9,10,11,12,13].

## 2. FcεRI Receptor Dynamics and Antigen Properties

**Figure 1:**
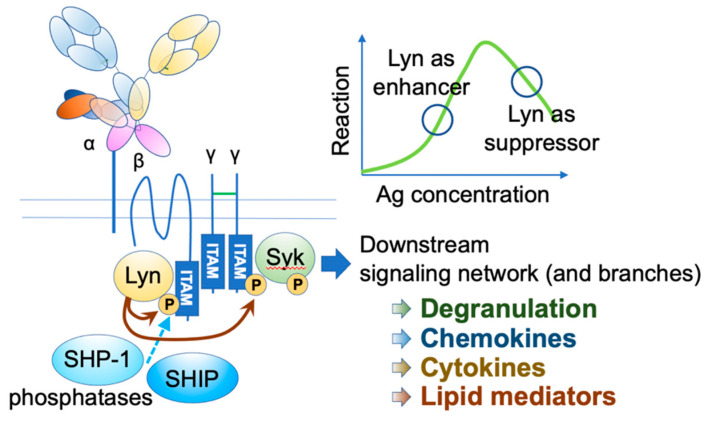
FcεRI activation. Upon FcεRI aggregation, Lyn initiates downstream signaling events through phosphorylation of ITAMs on FcεRI β and γ. Syk binds phosphorylated γ ITAM, and Lyn phosphorylates tyrosine residue of FcεRIγ-bound Syk. Activated Lyn and Syk further phosphorylate many downstream signaling molecules, leading to degranulation and de-novo synthesis of chemokines, cytokines, and lipid mediators. Under strong stimuli, Lyn recruits SHIP1 and SHP-1 via the phosphorylated FcεRI β ITAM and suppresses the reaction.

IgE functions through its receptors. The high-affinity receptor FcεRI is a heterotetramer, comprising an IgE-binding α chain, a signal-amplifying β chain, and two signal-initiating γ chains [4,5,6] (Figure 1). The β and γ chains have immunoreceptor tyrosine-based activation motifs (ITAMs) in their intracellular domains. Upon the engagement of multivalent antigens on FcεRI-bound IgEs, these receptors aggregate on the mast cell surface, and Lyn initiates the activation cascade by phosphorylating ITAMs, leading to a wide range of events, including degranulation, de novo lipid mediator synthesis, cytokine and chemokine production, as well as chemotaxis and survival of mast cells.

Human FcεRI has another assembly of αγ2 chains, which is expressed in several cell types, including dendritic cells (DCs). It plays a role in facilitating antigen presentation [14,15]. Although the αγ2 receptor is signaling competent, the co-existence of β chain and associated Lyn in mast cells significantly augments the signaling [16]. Furthermore, under the high intensity of stimuli, Lyn recruits two phosphatases, namely, Src homology 2 (SH2) domain-containing inositol polyphosphate 5-phosphatase 1 (SHIP1) and SH2 domain-containing protein tyrosine phosphatase 1 (SHP-1), through the phosphorylation of β chain ITAM, thereby providing safety mechanisms [17]. Lyn phosphorylation level is also fine-tuned by the adaptor function of phospholipase C-β3 (PLC-β3), which constitutively binds to SHP-1 [18].

**Figure 2:**
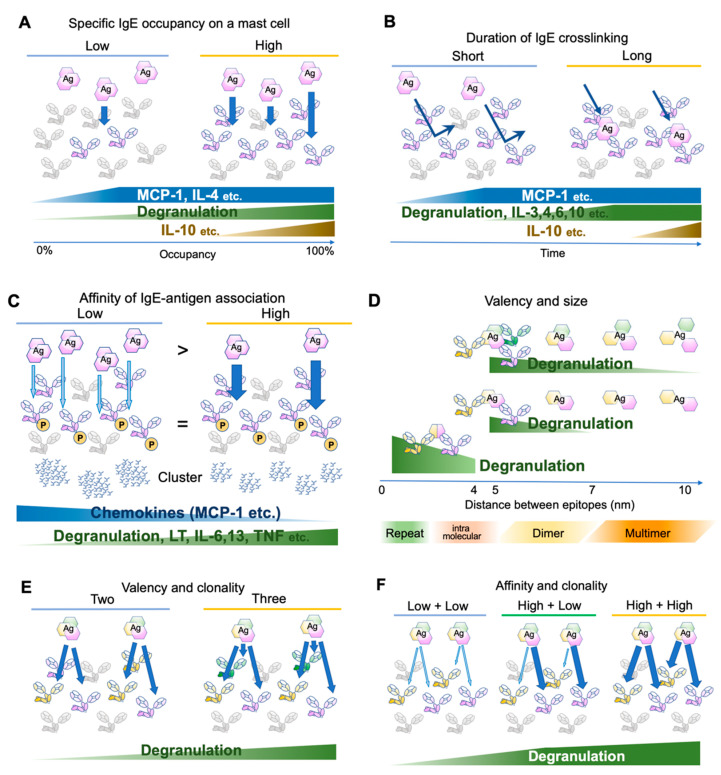
Differential mast cell activation by various antigens. (**A**) Effects of antigen-specific IgE occupancy on mast cell surface FcεRIs. (**B**) Effects of antigen-binding durations. (**C**) Effects of affinity between IgE and its cognate antigens. The antigen concentration is adjusted so that the phosphorylation levels of FcεRIβ and γ chains are similar. Abundant low-affinity antigens induce few and large clusters, while a small amount of high-affinity antigens induce many small clusters. (**D**) Effects of spacing between antigen epitopes. (**E**) Effects of antigen valency and/or IgE clonality. (**F**) Effects of affinity of different clones against the same antigen.

An early study has determined the number of FcεRI expressed on the rat peritoneal mast cells as approximately 3 × 10^5^ receptors per cell at the steady state [19]. Considering that the crosslinking of 100–1000 receptors is required for mast cell activation [20,21], theoretically a mast cell can be armed with IgEs for 300–3000 antigens at the same time. This number is concordant with a recent observation that 0.3% of antigen-specific IgE occupancy is required for the activation of human mast cells [22]. However, the activation consequences, such as degranulation and cytokine/chemokine production, are dependent on the receptor occupancy [23]. For instance, monocyte chemoattractant protein (MCP)-1 and interleukin (IL)-4 require only small occupancy, degranulation is almost linear to the occupancy, and IL-10 requires high occupancy [23] (Figure 2A). Because IgE may be produced locally in the tissue, different composition of antigen-specific IgEs on tissue mast cell surface may result in different consequences.

An important variable of the naturally occurring IgEs is the heterogeneity of the poly-clonal antibodies with different affinities against their target antigens. In addition, cross-reactivity between different antigens has been reported in closely related antigens from different species (e.g., seed allergens [24]) and completely different molecules with similar modification epitopes (e.g., carbohydrate allergens [25]). Polyreactivity of monoclonal IgEs has also been reported [26]. Furthermore, nonspecific binding may be observed at high but still in the physiological range of concentrations (~1000 IU/mL = 2400 ng/mL) [27]. Therefore, it is natural to ask whether IgE-sensitized mast cells can distinguish antigens with various parameters, such as affinity, valency, size, and contact duration. We will focus on these physical aspects of the antigen stimulation hereafter. The diversity of IgE-binding antigens, including autoantigen, has been covered by excellent reviews [12,28,29,30].

Several studies have investigated FcεRI distribution and the proximal signaling events [22,23,31,32,33,34,35,36]. Upon clustering, the FcεRI β and γ chain ITAMs are phosphorylated within 5 s [4,37], initiating the signaling cascade. Gonzalez-Epinosa et al. attempted to disassemble the clusters by adding monovalent haptens and examined the effect of clustering duration. Interestingly, they found three classes of actions: chemokines that rapidly become independent of the clustering (MCP-1, etc., 1–4 min after antigen application), degranulation and cytokines which require 4–8 min (IL-3, -4, -6, -10, etc.), and cytokines requiring more than 15 min (IL-2, -13, TNF-α, and IFN-γ) (Figure 2B). Moreover, MAP kinase phosphorylation substantially differs in the required clustering duration, suggesting the existence of distinct temporal thresholds for each signaling branch [23].

In an early study, Torigoe et al. used two related antigens, 2,4-dinitro-phenyl (DNP) and 2-nitrophenyl (NP) to investigate whether IgE-sensitization can distinguish between antigens with different affinities. The affinity of anti-DNP IgE toward NP moiety is approximately 1/1000 of the original affinity toward DNP. Using these antigens, the authors adjusted the NP antigen concentration such that FcεRI β and γ phosphorylation is more than that in DNP-stimulated cells. However, NP-induced degranulation was only one-tenth of DNP-induced degranulation [31]. Later, more careful adjustment was made to compare the consequences of the differences in affinity. Suzuki et al. stimulated anti-DNP IgE-sensitized bone marrow-derived mast cells with a series of DNP and NP concentrations but with exactly the same FcεRI β and γ phosphorylation levels. In addition to degranulation, lipid mediator release and cytokine/chemokine production were monitored. Intriguingly, low-affinity stimulation induced more chemokine production compared to high-affinity stimulation, while poor induction of degranulation, lipid mediator release, and cytokine production were observed [32] (Figure 2C). The authors further investigated the distribution of antigen-bound IgE and found that the abundant low-affinity antigens formed fewer but larger clusters than the high-affinity antigens did. The signaling events underneath the receptor shifted from the LAT1-dependent pathway to the LAT2 pathway, which was shown to contribute more to the chemokine production. Therefore, the affinity difference was physically converted to the spatiotemporal distribution of FcεRI, leading to the differential production of cytokines/chemokines. Consequently, the NP injection to the IgE-sensitized ear skin predominantly recruited monocytes and macrophages, while DNP recruited neutrophils [32]. It is tempting to speculate that such differential immune cell recruitment could contribute to the surveillance of newly emerging low-affinity antigens.

The clustering of FcεRI is cardinal to its activation. It seems that the cluster size is more dependent on soluble antigen concentration than its affinity [32,33]. Large cluster formation leads to receptor immobility and internalization, independent of the Src family kinase activity [33]. On the contrary, when mast cells encounter immobilized antigens of the micrometer order, the receptor and the proximal signaling molecules accumulate on the antigen-touched surface [34,38]. Furthermore, depending on the vertical distance between the cell and the target surfaces, the exclusion of the membrane-tethered CD45 phosphatase may occur and influence the activation strength [38]. This setting may mimic a situation in which the mast cells are touched by large intruders, such as nematodes. Importantly, Syk and the receptor-associating Lyn do not require F-actin polymerization for colocalization, while it is involved in the recruitment of LAT and PLCγ1 [34].

Anti-IgE monoclonal antibodies effectively crosslink IgE and activate mast cells, suggesting that the valency of two is sufficient for the cognate antigen to form clusters. However, the antigen concentration required for activation decreases with the increase in valency [33]. In addition, valency affects effector B cell responses [39]. Natural antigens often consist of dimers or oligomers [28] or repetitive sequences [29,40]. The term “allergen-associated molecular patterns” has been proposed to refer to these aspects of the antigen [29]. Compared to that of a synthetic antigen prepared by randomly conjugating haptens to a carrier protein (such as DNP25-BSA), for a natural antigen, one can assume that the major epitopes of oligomeric antigens are located at a fixed distance and orientation. To assess the effect of the distances between IgE binding epitopes, bi- or tri-valent antigens with a rigid scaffold of various lengths were tested for activation potency [35,36]. In both cases, the shortest scaffold of 4–5 nm provoked the highest activation, with gradually decreased activation up to 7 nm for bivalent antigens and 10 nm for trivalent antigens. As a reference, the maximum length of the lipocalin dimer (animal or insect-derived allergen family) is approximately 6–7 nm [41]. Therefore, the sizes of natural allergen dimer sizes may be in the range that can efficiently activate IgE-bound mast cells. In addition, the spacing of the same epitopes repeated intramolecularly at a distance of <4 nm was assessed recently in vitro and in vivo [42]. The results suggest that the shorter distances favor activation even in this range (~3.7 nm < ~2.6 nm < direct repeat, in PDB accession 1WLA), highlighting the role of repetitive sequences in efficiently provoking allergic responses [29,42] (Figure 2D).

Finally, Hjort et al. reported the effects of the clonality of IgEs with various combinations of affinities to a natural antigen [22]. In concordance with the previous reports showing that the valency is important for the mast cell activation [33,36], the combinations of three IgE clones for the same antigens were more effective than the combination of two in activating mast cells [22] (Figure 2E). Intriguingly, when two clones with different affinities were combined, the highest affinity among them was the major determinant of the kinetics (Figure 2F). Thus, the affinity maturation of IgE to one major epitope may be sufficient to reduce the activation threshold for the specific allergen [22].

## 3. Histamine-Releasing Factor (HRF)

HRF is a ubiquitously expressed protein cloned as a factor with an IgE-dependent histamine-releasing activity on human basophils [43,44]. It is coincidentally an essential intracellular protein for cell survival, proliferation, and malignant transformation in a variety of cell types [45,46,47]. In the latter context, HRF is called translationally controlled tumor protein (TCTP), fortilin, p21, and p23. Despite the lack of a canonical signal sequence, it can be secreted and found in the body fluids in atopic patients [43].

Interestingly, the prerequisite for basophils to respond to HRF is sensitization with IgE from certain atopic individuals [48,49]. Those IgEs that can prime basophils were termed IgE+, and the IgEs from healthy or myeloma donors that cannot do so were classified as IgE− [50]. However, the observation that the human FcεRI-expressing RBL mast cell line did not respond to HRF after sensitization with IgE+ led to a conclusion that HRF does not bind IgE+ [51].

Nevertheless, Kashiwakura et al. reasoned that IgE may have heterogeneity in terms of HRF reactivity and tested various IgE clones. Indeed, approximately 30% of the IgE clones were found to bind HRF [52]. The HRF-binding Igs were also found at a similar frequency in the IgG clones. Although the binding sites were mapped to the Fab region of Igs, the reactivity was not dependent on the antigen specificity. On the HRF side, N-terminal β-sheets consisting of 19 amino acids (N19) and the H3 helix were determined as the binding sites [52].

In the crystal structure of HRF dimer, which is disulfide-bonded at the C-terminal end C172, the distance between the N19 and H3 regions was approximately 4 nm, while the distance between the two H3 regions was 6 nm [53]. Although the structural basis for the requirement of both N19 and H3 regions to bind IgE remains unclear, the distances measured here are in the activation range for FcεRI crosslinking mentioned above, even if both binding sites in IgE are in close proximity to the antigen-binding sites.

The N19 or H3 fragment of HRF, or the HRF-2CA (a monomeric mutant of HRF, in which two cysteines are replaced by alanine) competitively inhibits the binding of HRF to the HRF-reactive IgE. Using these HRF inhibitors, HRF was shown to play a role in IgE-dependent allergic inflammation in the skin, the airway, and the intestine [52,54,55]. In the food-allergic mice, HRF dimer/oligomers were produced in the intestine, and serum levels of HRF-reactive IgE were increased. Intragastric administration of HRF inhibitors significantly reduced diarrhea occurrence and body temperature drop, in both preventive and therapeutic contexts. In humans, patients allergic to hen’s eggs also had elevated HRF-reactive IgE levels, and the successful oral immunotherapy induced a sustained reduction of HRF-reactive IgEs [54,55]. These observations suggest that the secreted HRF dimer/oligomers bind HRF-reactive IgEs and promote the antigen-induced crosslinking of FcεRI on mast cells and basophils, leading to food allergy elicitation.

Besides patients with food allergy, patients with atopic dermatitis (AD) [26], chronic spontaneous urticaria (CSU) [56,57], pulmonary arterial hypertension [58,59], and acute liver rejection [60] have elevated HRF levels in circulation. Elevated levels of HRF-reactive IgEs have also been detected in patients with AD and CSU [26,56].

N-terminally truncated HRF dimers have been reported to have another cytokine-like function exerted through its flexible loop and helix H2 [61,62]. This activity is reasonably IgE independent, since it lacks N19 binding sites [57]. However, the receptor has not been identified for this function, and whether the truncated HRF can be internalized remains unknown. In addition, HRF-containing exosomes may have intracellular functions in target cells [58,59]. Therefore, care should be taken when the IgE-dependent extracellular roles of HRF are examined separately from others. In any case, monomeric HRF-2CA may be a good candidate for the drug targeting HRF.

## 4. IgE Structural Conformation

**Figure 3:**
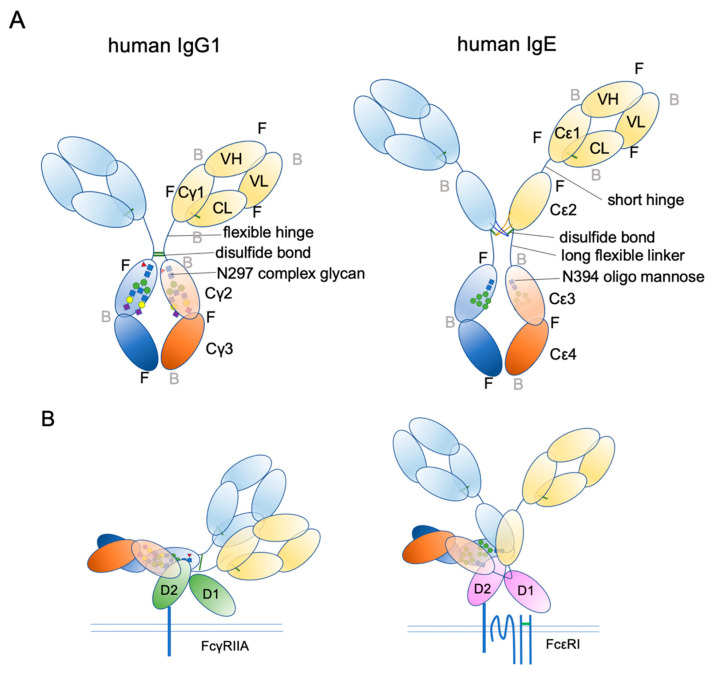
Schematic representation of human IgG1 and IgE. (**A**) The extended conformations. The three-dimensional coordination (front or back) is shown. F, front; B, back. (**B**) The receptor bound conformations. Please note that IgE Fab is almost in the upright position, suitable for sensing its target antigens.

**Figure 4:**
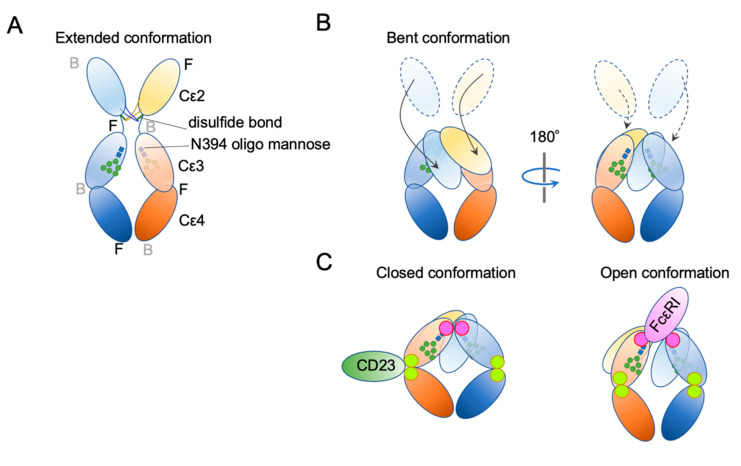
Schematic representation of two major conformational concepts. (**A**) An extended conformation of IgE-Fc. The front–back coordination is also described. (**B**) The bent conformation. (**C**) The concept of “closed” and “open” conformations.

Ig structure is often depicted as a Y-character-like scheme with two heavy chains and two light chains (Figure 3). This schematic representation may give the impression that Igs are made of two mirror-image arms. However, considering that the two arms have the identical peptide chains made of L-forms of amino acids, it cannot be true; it rather has a two-fold rotational symmetry if at all. In reality, the flexible hinge between the Fab and Fc regions allows for various angles of Y shapes, thereby providing considerable latitude in the distance and the angle for binding two antigens [63]. In IgE, things are more complicated. The hinge region is shorter than that of IgG and seems to have less flexibility [64]. Instead, the Fc portion of IgE can be acutely and asymmetrically bent (Figure 4A,B) [65,66,67], and an appropriate bend is required for the binding to FcεRI [64,68,69]. Interestingly, an antibody Fab fragment called aεFab can straighten this bend by binding from two sides at high concentrations and block IgE’s binding to FcεRI allosterically [67]. Unlike IgG subclasses with three Ig-like domains in the constant region, IgE has a longer tail, composed of four Ig-like domains, of which Cε2 has no counterpart in IgGs. The bend occurs at the long linker region between Cε2 and Cε3, and the exposed N-terminal sides of two Cε3 engage the FcεRIα chain at different sites on both molecules (Figure 4B,C) [68,70]. It was shown that the bent form of free IgE-Fc is the most stable in terms of the free energy level [67]. However, another recent negative stain electron microscopy study suggested a major free IgE conformation with less bent Cε2 and asymmetrically oriented two Fabs [64]. Since this conformation is incompatible with FcεRI binding, transient bending may be required for FcεRI engagement. Upon binding to the FcεRI with one side of Cε3 in bent conformation, a minor alteration of intramolecular conformation occurs within Cε2 and Cε3, leading to the full engagement of IgE to FcεRIα [68]. Although Cε2 does not have direct contact with FcεRIα, it contacts with Cε3, and stabilizes the binding by slowing the dissociation rate from FcεRIα (koff ~ 10^−5^ s^−1^) [71,72]. The estimated association constant Ka of IgE and FcεRIα is approximately 1 × 10^10^ M^−1^, which is in the order of Ka for antibody-antigen binding [71]. Tight binding with an exceptionally slow dissociation rate is considered a key property of IgE-FcεRI association that allows for a long half-life in the peripheral tissue.

In addition to bending, another important conformational concept of IgE is “closed” and “open” forms (Figure 4C). IgE has another, but so-called low-affinity receptor, CD23, also known as FcεRII. CD23 is a type II transmembrane protein that exists as a homotrimer. Its IgE-binding C-type lectin-like domain is located in the extracellular C-terminal head. Although the affinity of a single head for IgE is estimated to be 10^6^–10^7^ M^−1^, the avidity of the trimer to IgE is considered to be close to that of FcεRI. Interestingly, the binding of CD23 and FcεRI to the IgE is mutually exclusive; the binding of CD23 to the hinge region of Cε3 and Cε4 allosterically close the binding site of FcεRI (closed form). Conversely, the binding of FcεRI (open form) to Cε3 makes the CD23 binding site inaccessible. This mutual exclusion mechanism ensures that the IgEs on cell surface FcεRI cannot be mistakenly crosslinked by cell–cell contact with CD23-expressing cells. It is also noteworthy that both soluble FcεRIα fragment [73,74,75] and soluble CD23 (sCD23) [76,77,78] may theoretically block IgE binding to both receptors. For more details on the roles of CD23 in allergic reactions, the reader is referred to the review article by Engeroff et al. [11].

## 5. IgE-Binding Therapeutics

**Figure 5:**
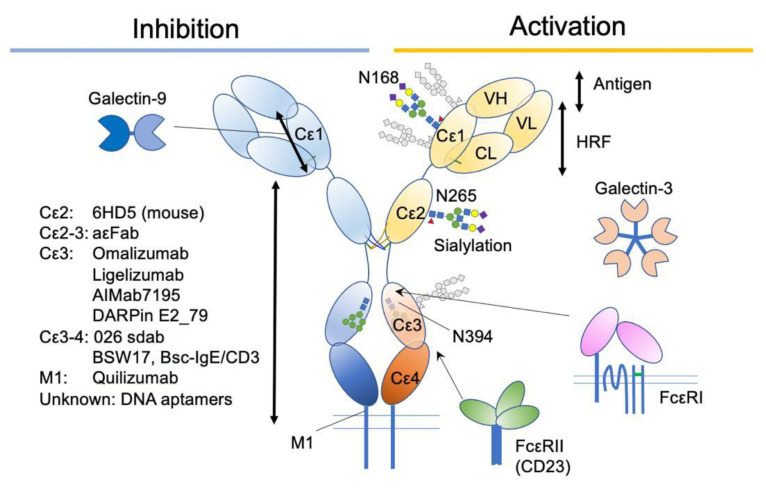
A summary of IgE-associating molecules and their binding sites.

Antibodies and other substances targeting IgE have been developed to treat allergic diseases (Figure 5). Omalizumab (Xolair^®^) is the first and most characterized. The efficacy of omalizumab is shown for many allergic diseases, including allergic asthma [79,80,81,82,83], chronic urticaria [84,85], nasal polyposis [86,87], and pollinosis [88], and approved for the treatment of moderate to severe persistent allergic asthma, chronic idiopathic urticaria (CIU) and nasal polyps in the USA. It is also approved for severe pollinosis in Japan. In addition, its benefit in an adjunctive use with immunotherapy has been reported, particularly in preventing the adverse reactions [89]. These results suggest that IgE plays a crucial role in the pathology of a certain range of allergic diseases.

Omalizumab is a recombinant humanized IgG1κ monoclonal antibody that specifically binds the Cε3 domain of human IgE. Omalizumab specifically binds free IgE (not bound to FcεRI) and blocks its binding to both FcεRI or CD23 receptors. Since it does not affect the FcεRI-bound IgEs, the reduction of IgE on mast cells takes longer (~70 days) than that on basophils (~7 days), depending on their half-lives [90,91]. The initial design of omalizumab was to bind the same site of IgE as FcεRI does, thereby preventing anaphylaxis caused by crosslinking the FcεRI-bound IgE. However, X-ray crystallographic studies have shown a slightly different view of the mechanisms.

It seems that the crystallization of the complex of IgE-Fc and omalizumab Fab requires some tweaking, such as constraining the Cε3-4 conformation to be “closed” by the introduction of an extra disulfide bond (G335C) [92], or inhibiting the favored crystallization of unbound Fab fragments by introducing three point mutations to disrupt contacts observed in omalizumab Fab crystals (FabXol3) [69]. Although the former strategy suggested that the steric hindrance inhibits omalizumab-bound IgE’s engagement to FcεRI [92], by nature it could not predict the effect of conformational change, including Cε2 bending. Contrary to the previous study, the latter strategy revealed that the allosteric effect is the major source of inhibiting activity of omalizumab [69]. However, binding to CD23 was shown to be inhibited by steric hindrance in both reports.

Interestingly, the allosteric effect exerted by omalizumab made the bent knees of Cε3 more open than that of FcεRI-bound conformation. In line with this, omalizumab can facilitate the dissociation of IgE from FcεRI, although at a very high concentration that cannot be achieved in the therapeutic use (50 μM = 7.5 mg/mL) [93].

An antibody Fab fragment aεFab described above binds mainly at the Cε3 portion of the human IgE. The bent form of free IgE exposes one binding site for aεFab, and this binding is enough for inhibiting the binding of FcεRI. In the bent form, it has steric hindrance with FcεRI, and in the extended form, it has an allosteric hindrance [67]. At higher concentrations (Kd = 1500 nM), the second aεFab binds from the opposite side, and straitens the whole IgE-Fc to make it almost at the symmetrical Y-shape.

MEDI4212 is another antibody against IgE, which underwent a clinical trial. MEDI4212 was developed from the parent IgG ENG085, which was selected as a human single-chain variable fragment by a large phage library. The X-ray co-crystallography with the Cε3-4 fragment showed that MEDI4212 binds the Cε3 portion and keeps it in an “open” conformation, suggesting that its mode of action is steric hindrance for FcεRI and allosteric hindrance for CD23 [94]. MEDI4212 showed much higher affinity than omalizumab to IgE [94] and quick removal of free IgE was observed in phase I trial [95]. However, the major shortcoming of MEDI4212 was the shorter half-life than that of omalizumab. Therefore, the rapid recovery of free IgE levels was observed [95].

Ligelizumab (QGE031) is also a humanized IgG1 monoclonal antibody that binds with higher affinity to the Cε3 domain of IgE than omalizumab does. Despite its failure in the treatment of severe asthma [96], it showed a superior therapeutic result in the treatment of CSU [97]. It has demonstrated a rapid and sustained reduction in the basophils surface IgE as well as FcεRI levels with a smaller dose than that of omalizumab [98]. The crystal structure of ligelizumab-bound Cε3-4 fragment showed that the major contact was ligelizumab VH and Cε3, with a minor contact of VL to another chain of Cε3, adjacent to N394 [99]. The contact mapping suggests that ligelizumab sterically blocks FcεRI engagement [99]. In addition, a negative electron microscopy revealed that the IgE in solution is bound by ligelizumab from two sides, constraining it in an extended conformation [64]. In contrast to omalizumab, ligelizumab did not show any dissociation activity on FcεRI-bound IgE [99].

Intriguingly, although the FcεRI binding inhibition potency of ligelizumab is higher than that of omalizumab, that on CD23 is lower compared to that of omalizumab [96,99]. From the crystallography, it was suggested that the minor overlap of ligelizumab and CD23 binding sites, and the allosteric effect to keep IgE in a relatively open conformation, are the major mechanisms for CD23 binding inhibition [99,100].

DARPins (designed ankyrin repeat proteins) are a relatively new class of synthetic proteins derived from natural ankyrin repeat proteins, which can be designed to have diverse binding specificities [101]. The DARPins against human IgE have been selected from a large library using ribosome display method [102]. Among them, DARPin E2_79 binds Cε3 from the outside of the binding sites for FcεRI and accelerate the dissociation of IgE from FcεRI by the “facilitated dissociation” mechanism [103,104]. “Facilitated dissociation” is an old-and-new view of competition between binding molecules, where the partially bound states of two competing molecules are considered, instead of taking account of only bound and unbound states [104,105,106]. In the case of DARPin E2_79, the small overlap of binding sites for DARPin E2_79 and FcεRI may allow for the partial and intermittent binding of both molecules. The competition at that small part is considered to accelerate the dissociation of FcεRI [103]. This example demonstrates the importance of small steric overlap, alongside allosteric effects, on the dissociation of IgE molecules from tightly bound FcεRI. Further improvement of displacement activity (by ~100 fold) was achieved by tandemly conjugating another IgE-binding DARPin to E2_79, aiming to increase avidity [93].

In addition to the Cε3 domain, the Cε3-4 region has been targeted. Single-domain antibody (sdab) 026 is a llama-derived, humanized sdab-targeting Cε3-4 region of human IgE [100]. The binding of 026 sdab constrains the Cε3-4 fragment at the “closed” conformation, mimicking the binding of CD23. In addition, synchrotron small-angle X-ray scattering (SAXS) observation, which allows for determination of the conformation of the flexible macromolecular complex in solution at low resolution [66], showed a slightly unbent and extended location of Cε2. These allosteric conformational changes block the engagement of IgE to FcεRI [100]. The most striking activity of 026 sdab is the displacement of IgE from both FcεRI and CD23. Despite the similar conformational constraint of IgE, the shared footprint as well as the steric overlap of 026 sdab and CD23 was relatively minor [100], suggesting that the “facilitated dissociation” can take place. In addition, one of the 026 sdab binding sites is accessible in the FcεRI-bound IgE. Therefore, it may be possible that allosteric conformational changes accelerate the dissociation of IgE from FcεRI.

In addition to Cε3 and Cε4, Hirano et al. recently reported that a Fab fragment (clone 6HD5)-binding murine Cε2 reduces mast cell activation in vivo and in vitro [107]. Interestingly, the suppressive effect on passive cutaneous anaphylaxis continued for up to 10 days after Fab injection. In addition, the effect was exerted without removing IgEs. The failure of the attempt to map the binding site inside Cε2 with the recombinant short fragments suggests that the binding site is conformational [107]. Although the precise mechanism underlying this suppression requires further investigation, the observation implies that there is another role for Cε2 other than stabilizing engagement to the FcεRI [72].

Another strategy to suppress IgE is to reduce production. Quilizumab is a humanized, afucosylated, monoclonal IgG1 antibody targeting the M1-prime segment present only in membrane-type IgE. It was intended to remove memory IgE B cells. Despite the successful reduction in antigen-specific and total IgEs by up to 40%, it failed to improve CSU [108] and inadequately controlled allergic asthma [109]. Interestingly, an antigen challenge-induced increase in IgE was not observed in quilizumab-treated patients, suggesting that quilizumab efficiently eliminated the new development of IgE-producing cells [110]. It has been speculated that long-lived IgE plasma cells lacking membranous expression of IgE may produce sufficient pathogenic IgEs [108,109].

AIMab7195 (former XmAb7195) is a humanized, modified IgG1 monoclonal antibody, with enhanced binding activity on FcγRIIb [111]. FcγRIIb is an Fc receptor expressed on B cells, basophils, and macrophages, and the crosslinking of B cell receptor (membrane-type of Igs) with FcγRIIb suppresses B cell activation [112]. Therefore, AIMab7195 is aimed to have two functions: sequestration of free IgEs and suppression of newly produced IgEs. However, the result of the phase I trial has not yet been published.

Bispecific IgE/CD3 antibody (bsc-IgE/CD3) has also been developed to eliminate IgE-producing B cells [113]. It was inspired from the successful development of bispecific anti-CD19/CD3 antibody called blinatumomab, which is used as an immunotherapy for B cell-derived Acute Lymphoblastic Leukemia [114,115,116]. Bsc-IgE/CD3 is composed of two single-chain variable fragments (scFv) against IgE and CD3, with a linker region in between. The anti-IgE scFv is derived from non-anaphylactogenic anti-IgE clone BSW17, which binds Cε3-4 domains but only those in the free IgE [117]. Bsc-IgE/CD3 connects membrane-type IgE-expressing cells with cytotoxic T cells, leading to specific cell lysis [113]. However, because of the conformational preference, IgE-bound cells on their FcεRI or CD23 were unaffected.

Other drug candidates targeting IgE include DNA aptamer [118]. However, the structural basis for antagonizing IgE binding to FcεRI has not been well characterized [119,120,121].

## 6. Glycosylation

IgE is a heavily glycosylated molecule, with seven N-glycosylation sites in human IgE and nine N-glycosylation sites in mouse IgE. To date, there are no known O-glycosylation sites in IgEs. Most of these sites have complex glycans, except for N394 in humans and N384 in mice, which are conjugated with oligomannose glycans [122,123]. Interestingly, this glycosylation site is conserved among mammalian IgEs, as well as in other Ig isotypes [124]. It corresponds to a key N-glycosylation site, N297, on the Cγ2 of human IgG1, although the attached glycans are fucosylated biantennary complex glycans (Figure 3A) [124,125,126]. As mentioned above, IgG lacks the Cε2 counterpart. Therefore, the Cγ2 of IgG1 corresponds to the Cε3 of IgE. The oligomannose at N394 has a core comprising two tandem N-acetylglucosamines (GlcNAc) and three mannose residues, making the first bifurcation. Several additional mannose residues follow the core structure. The complex glycan chains at other sites are attached by extra sugar residues, such as a fucose at the first GlcNAc in the core, and additional GlcNAc, galactose, N-acetylgalactosamine (GalNAc), and sialic acids after a few bifurcations by mannose residues, making bi-/tri-/tetra-antennary structures [68,123,124,126,127,128].

The functions of these glycosylations have been extensively studied using glycan digestive enzymes and recombinant proteins produced in *Escherichia coli* (aglycosylated) and mammalian cells (glycosylated). These studies primarily focused on their effects on FcεRI binding. Despite the inconsistent results reported in earlier studies, recent mutational studies on each N-glycan site of whole IgE expressed in mammalian cells have concluded the absolute requirement of N394 glycosylation for binding to FcεRI [100,123,124]. The direct substitution of N394 with threonine (N394T) [124], glutamine (N394Q) [100,123], and the disruption of the N-glycosylation site (Asn-X-Ser/Thr) by mutating the third amino acid to alanine (T396A) [123] abolish mast cell activation in vitro and in vivo. Furthermore, the mouse counterpart N384 is also essential for binding to FcεRI [123], suggesting that its role in maintaining IgE conformation is shared by mammals. An experiment leaving only the N384 glycan site intact, but disrupting all other sites on mouse IgE, showed that the N384 glycan is sufficient for binding to FcεRI [123].

The structural basis for the role of the N394 glycan remains obscure. A crystallography of a human IgE fragment detected the first GlcNAc on N394 glycan at the interface of Cε2 and Cε3 and a potential hydrogen bond with D271 on Cε2 [68]. In this report, the rest of the glycan chain was disordered and could not be determined. In mice, human D271 is conserved as D261. An endoglycosidase Endo F1 cleaves N-glycans at the linkage between the first and second GlcNAcs, thereby leaving the first GlcNAc in place. The observation that the Endo F1 treatment of mouse IgE abolished FcεRI binding [123] suggests that the first GlcNAc is not sufficient to hold the proper conformation of IgE to bind FcεRI. Further investigation is required to elucidate the mode of action of the N394 glycans.

Although these enzymatic and mutational strategies can introduce all-or-none modifications of desired N-glycans, the glycan composition is heterogenous and subject to change in health and disease conditions. In addition, it is well established that the composition of the N297 glycan on human IgG1 determines its functional consequences [129]. A recent study by Shade et al. [130] reported alterations of the glycan composition in allergic individuals. They found more sialylation, and therefore less galactose exposure, on the N265 glycan in allergic patients. Similar results were observed for N140 and N168. The function of sialylation was tested on human and mouse IgE in vivo and in vitro, and asialylation reduced the potency of IgE to induce degranulation, without altering the affinities against FcεRI and antigens [130]. Interestingly, not only the asialylation of IgE but also the co-existence of asialylated glycoproteins attenuated mast cell activation. Although the precise mechanism is yet to be reported, the exposed terminal structures, including galactose, might exert a suppressive function through binding to galectins or other galactose-binding proteins (see below).

## 7. Galectins

Galectins are a family of β-galactoside-binding lectins with 15 members in mammals [131]. They are classified into three groups based on the number of carbohydrate recognition domains (CRDs) and their terminal structure; (1) the single-CRD prototypic galectins, which can be assembled into homodimers by non-covalent bindings (Gal-1, Gal-2, Gal-5, Gal-7, Gal-10, Gal-11, Gal-13, Gal-14 and Gal-15), (2) tandem repeats of two distinct CRD domains connected by a linker region (Gal-4, Gal-6, Gal-8, Gal-9 and Gal-12), and (3) a “chimera” type of galectin (Gal-3) composed of a single C-terminal CRD, a collagen-like linker, and an N-terminal domain, which plays a role in oligomer (up to pentamer) formation and protein binding [131,132]. Galectins lack signal sequences and are secreted by an unconventional pathway after their synthesis in the cytoplasm [131,132,133].

Galectin-3 (Gal-3) was first cloned as an IgE-binding molecule [134] and was identified as a macrophage-derived protein mac-2 [135]. It is associated with various human diseases, including allergy, autoimmune diseases, cancer, and cardiovascular diseases [132]. Recently, it was reported that Gal-3, secreted and bound on the surface of epithelial cells, can crosslink IgEs on the sensitized basophils and stimulate IL-4/13 release in vitro [136]. Since Gal-3 oligomerizes and becomes immobilized on the cell surface at the physiological concentration [137], this observation suggests that the β-galactoside binding capacity of the oligomerized Gal-3 in the galectin lattice is not saturated. However, it seems difficult to dissect this effect from the anti-inflammatory effects in vivo [138,139,140,141]. One way to address this issue would be the manipulation of glycans on IgE chains, since this strategy would not affect a wide range of intracellular and extracellular Gal-3 functions [131,132,141].

Gal-9 is another galectin that binds to glycans on the IgE molecule [142]. Gal-9-bound IgE loses affinity to its specific antigen. Furthermore, the suppression of IgE-dependent mast cell activation was shown in vivo by the attenuation of the passive cutaneous anaphylaxis model, as well as in vitro by the inhibition of degranulation and calcium mobilization [142]. Because mast cells express a high amount of Gal-9, and their expression increases by antigen plus IgE stimulation, it was speculated that it may serve as a feedback mechanism. Although Gal-9 binds to both human and mouse IgEs, the responsible glycan sites have not been elucidated. Furthermore, whether the suppressive effect on antigen binding is specific to the tested IgE clone (SPE-7) or applicable to any IgE is unknown. Considering the report that the mutational disruption of all three N-linked glycosylation sites in Cε1 led to slightly reduced IgE-mediated degranulation [123], Gal-9 might recognize the glycans close to antigen-binding sites to tweak the antigen-binding strength.

## 8. Concluding Remarks

During the past 55 years, since the discovery of IgE, the development of new technologies has enabled detailed analysis of a structural, physicochemical, and molecular biological basis for the IgE-dependent activation of mast cells. The efforts to elucidate the true nature of IgE have revealed that this single molecule, in fact, has a diversity; the conformation is flexible, the glycosylation may change in health and diseases, and the reactivity to antigens and IgE-interacting molecules such as histamine-releasing factor may vary. The heterogeneity of IgE and their ligand antigens are sensed via FcεRI and translated into a variety of modes of activation in mast cells. A recent report demonstrated that IgE-antigen immune complexes are less bound by FcεRI and sequestered through CD23 binding, whereas the free IgE is preferred by FcεRI [143]. This is in line with the notion that IgE is provided to function as a sensor, but not for neutralization of the antigens. Tuning the sensor is vital to its proper function. Thus, the appropriate use of emerging IgE-binding therapeutics to tweak the functions of IgE may provide us opportunities to treat various allergic diseases.

## Data Availability

Not applicable.
